# Effects of dietary inulin, statin, and their co-treatment on hyperlipidemia, hepatic steatosis and changes in drug-metabolizing enzymes in rats fed a high-fat and high-sucrose diet

**DOI:** 10.1186/1743-7075-9-23

**Published:** 2012-03-27

**Authors:** Junko Sugatani, Satoshi Sadamitsu, Tadashi Wada, Yasuhiro Yamazaki, Akira Ikari, Masao Miwa

**Affiliations:** 1 Department of Pharmaco-Biochemistry, School of Pharmaceutical Sciences, University of Shizuoka, 52-1 Yada, Surugaku, Shizuoka City, Shizuoka 422-8526, Japan; 2 Global Center of Excellence for Innovation in Human Health Sciences, School of Pharmaceutical Sciences, University of Shizuoka, 52-1 Yada, Surugaku, Shizuoka City, Shizuoka 422-8526, Japan; 3 Fuji Nihon Seito Corporation, 1-4-10 Shimizuseikai, Shizuoka City, Shizuoka 424-8737, Japan

**Keywords:** Synthetic inulin, Hepatic steatosis, CYP1A1/2, CYP2E1, Lipid-lowering drug, Fluvastatin

## Abstract

**Background:**

Rats fed a high-fat and high-sucrose (HF) diet develop hepatic steatosis and hyperlipidemia. There are several reports that a change in nutritional status affects hepatic levels of drug-metabolizing enzymes. Synthetic inulin is a dietary component that completely evades glucide digestion. Supplementing a HF diet with inulin ameliorates hypertriglycemia and hepatic steatosis, but not hypercholesterolemia. This study aimed at distinguishing the effects of synthetic inulin and 3-hydroxy-3-methylglutaryl coenzyme A reductase inhibitor (statin), which inhibit cholesterol biosynthesis.

**Methods:**

We examined effects of co-treatment with synthetic inulin (5%) and fluvastatin (0, 4, and 8 mg/kg, *per os*) on body weight, epidydimal white adipose tissue weight, serum and hepatic lipid profiles, and hepatic cytochrome P450 (CYP) mRNA and protein profiles in rats fed a standard diet or a HF diet for 3 weeks.

**Results:**

Treatment with the synthetic inulin (5%) or fluvastatin at 4 mg/kg (lethal dose in rats fed the HF diet, 8 mg/kg) ameliorated the elevation in hepatic triacylglycerol and total cholesterol levels in rats fed the HF diet. Whereas co-treatment with the inulin (5%) and fluvastatin (4 mg/kg) had a tendency to more strongly suppress the elevation in serum levels of very low density lipoprotein triacylglycerol than either treatment alone, no additive or synergistic effect was found in decrease in hepatic lipid levels. Hepatic levels of CYP1A1/2 and CYP2E1 mRNA and protein and methoxyresorufin *O*-demethylase and ethoxyresorufin *O*-deethylase activities were reduced in rats fed the HF diet. The synthetic inulin alleviated the reduction in hepatic levels of CYP1A1/2 and CYP2E1 mRNA and protein more strongly than fluvastatin, and no synergistic effects were observed on co-treatment. Furthermore, hepatic levels of aryl hydrocarbon receptor mRNA were decreased in rats fed the HF diet and recovered to near normal values with the intake of dietary inulin, which correlated with change in CYP1A1/2.

**Conclusions:**

Dietary inulin alone was effective to prevent the development of hepatic steatosis, ameliorate nutritional effects, and alleviate the hepatic change in the expression of CYP1A1/2 and CYP2E1, while co-treatment with statin did not have additive or synergistic effects and statin may cause adverse effects in rats fed the HF diet.

## Background

The liver plays a central role in metabolizing therapeutic drugs and environmental contaminants. The activities of drug-metabolizing phase I and II enzymes in the body are affected by the genotypes of the translating gene and also by non-genetic factors including environmental factors. The expression of cytochrome P450 (CYP) 2E1, a microsomal oxidase involved with fatty acid ω-oxidation, as well as CYP4A, is upregulated during starvation, fasting, obesity and hyperlipidemia [1-4]. Elevated levels of CYP2E1 have been largely attributed to the pathogenesis of liver disease in patients with nonalcoholic steatohepatitis (NASH) [3-7]. In contrast, Fisher et al. [8] reported that the expression of CYP2E1 significantly decreased with the progression of human nonalcoholic fatty liver disease (NAFLD) from simple fatty liver (steatosis) to the more severe NASH, and the expression of CYP1A2, CYP2C19, CYP2B6 and CYP3A4 also tended to decrease with increasing severity of NAFLD. These observations were not consistent with reports of increased CYP2E1 expression in livers from patients with NAFLD [9,10]. Although nutritional factors such as starvation, fasting and a high-lipid diet have been reported to modulate liver microsomal CYP composition, leading to the altered hepatic metabolism of drugs, carcinogens, steroid hormones, and fatty acids, little is known about whether the suppression of lipid accumulation in fatty liver alleviates the changes in hepatic CYP composition. Thus, it is worth investigating how to suppress the changes in hepatic CYP composition associated with hepatic steatosis, which is proposed to be the setting for more severe liver diseases such as nonalcoholic steatohepatitis with histologic signs of fibrosis and necroinflammation through to cirrhosis, terminal liver failure and hepatocellular carcinoma [11].

Some dietary components that completely evade digestion, such as resistant starch and inulin, have been demonstrated to exert systemic effects by modifying lipid metabolism [12-14]. Previously [15], we reported *Bacillus sp. 217 C-1 *expressing a highly efficient enzyme that converts sucrose into inulin molecules, which comprise a linear polymer linked by β(2 - 1) glycoside bridges of D-fructose with one terminal glucose similar to plant-derived inulin and have a similar property for *in vitro *fermentation to plant-derived inulin and suppress the growth of harmful bacteria more strongly than fructooligosaccharide [14-17]. Feeding a high-fat and high-sucrose (HF) diet to rats for 8 to 12 weeks produced hyperlipidemia and hepatic steatosis, and supplementing the diet with the synthetic inulin reduced the elevation in body weight, epidydimal white adipose tissue (WAT) weight, and serum and hepatic levels of triacylglycerols [15,18]. Thus, in this study, we characterized the changes in expression of CYP mRNA and protein associated with alterations in nutritional status such as serum and hepatic lipid profiles. Statin, 3-hydroxy-3-methylglutaryl coenzyme A (HMG-CoA) reductase inhibitor, whose competitive inhibition of HMG-CoA reductase reduces the amount of HMG-CoA converted to mevalonate, the rate-limiting step of cholesterol biosynthesis, is a member of an important class of lipid-lowering drugs. Since the synthetic inulin did not suppress the elevation in serum cholesterol levels of rats fed a HF diet, we investigated the influence of co-treatment with the synthetic inulin and fluvastatin on the health of rats fed a HF diet and the changes in hepatic CYP expression caused by fatty liver.

## Methods

### Chemicals

Inulin [average degree of fructose polymerization (DP) = 16 ~ 17] enzymatically synthesized from sucrose by an inulin-producing enzyme was prepared as reported previously [15]. Fluvastatin was purchased from Toronto Research Chemicals, Inc. (North Yolk, Canada).

### Experimental animals

All experiments followed protocols approved by the Institutional Animal Care and Life Committee, University of Shizuoka. Male Wistar rats were obtained from Japan Charles River (Tokyo, Japan) at 6 weeks of age. Animals were acclimatized for one week prior to the experiment, housed in stainless-steel hanging cages with free access to food and water, and maintained on a 12-h light-dark cycle. All animals were randomly assigned to the standard (SD) diet, 5% inulin-supplemented standard (SD + I) diet, high-fat and high-sucrose (HF) diet, or 5% inulin-supplemented high-fat and high-sucrose (HF + I) diet [17-19]. After one week on either diet, each group was divided into three subgroups and given 0, 4, or 8 mg fluvastatin/kg/day (*per os*) (4 rats/group) since the lethal dose of fluvastatin in SD diet- and HF diet-fed male rats was 16 mg/kg/day and 8 mg/kg/day, respectively, with the diet for 2 weeks [20]. The HF diet consisted of 19.7% casein, 1% soybean oil, 10% lard, 4% mineral mixture, 1% vitamin mixture, 0.15% choline chloride, 0.5% cholesterol, 0.25% sodium cholate, 3.4% cellulose and 60% sucrose (23.9% lipid, 56.8% carbohydrate and 19.3% protein [kJ]). The SD diet consisted of 23.8% crude protein, 5.1% crude fat, 3.2% crude fiber, 6.1% ash, 54% nitrogen-free extract and 7.8% humidity (12.9% lipid, 60.4% carbohydrate and 26.7% protein [kJ]). The rats were weighed three times per week, and food intake in grams was monitored. Each experiment was done at least twice.

### Blood and tissue sampling

Rats about 4 h after removal of the diets unless otherwise stated were anesthetized with diethyl ether, the abdominal cavity was rapidly opened, and blood was drawn from the abdominal vena cava or portal vein into syringes between 11:00 AM and 12:00 PM. To prepare fasting animals, at the end of experimental period, the groups were starved for 16 h before being sacrificed. Plasma samples were separated from blood collected into heparinized tubes by centrifugation, and serum samples were separated from blood by centrifugation after standing for 30 min at room temperature. The resulting plasma/serum was stored at -80°C prior to analysis. Livers were rapidly excised and weighed. The liver median lobe was excised for the preparation of microsomes, plasma membranes and nuclear extracts and the extraction of RNA.

### Biochemical analyses

Blood and tissue sampling was done as described previously [19]. Portal plasma glucose concentrations were determined by the hexokinase method using commercial reagents (R-Biopharm AG, Darmstadt, Germany). Frozen livers (about 0.5 g) were homogenized in 20 volumes (the SD group) or 100 volumes (the HF group) of 0.9% NaCl containing 0.1% Triton X-100, and triacylglycerol and total cholesterol concentrations in serum and liver were estimated with kits from Shino Test (Tokyo, Japan).

Triacylglycerol and total cholesterol levels in serum lipoproteins were determined by a dual detection HPLC system with two tandemly connected TSKgel Lipopropak XL columns (300 × 7.8 mm; TOSOH) in Skylight Biotech Inc. (Akita, Japan) [17].

### Preparation of microsomes

Liver microsomes were prepared by differential centrifugation, first at 9,000 g for 15 min then at 105,000 g for 60 min, at 4°C and stored at -80°C. The microsomal protein concentration was determined with a bicinchoninic acid protein assay kit (Pierce Chemical, Rockford, IL) using bovine serum albumin as a standard.

### Immunoblot analysis

Microsomal proteins (20 μg) were resolved on a sodium dodecyl sulfate-12.5% polyacrylamide gel, and electroblotted onto a polyvinylidene difluoride membrane (Millipore Corporation, Bedford MA). The immunoblots were incubated with primary antibodies against rat CYP1A1 (the antibody also recognizes rat CYP1A2, whose molecular weight is lower than that of rat CYP1A1) (dilution 1:10,000), rat CYP2B1 (dilution 1:10,000), rat CYP2C11 (dilution 1:10,000), rat CYP2E1 (dilution 1:5,000), rat CYP3A2 (dilution 1:10,000), rat CYP4A1 (dilution 1:10,000), and rat NADPH P450 reductase (CPR) (dilution 1:10,000) (Daiichi Pure Chemicals Co., Tokyo, Japan). Antigen-antibody complexes were detected using the appropriate secondary antibody conjugated with horseradish peroxidase and visualized with an enhanced chemiluminescence system (GE Healthcare Bio-Sciences, Piscataway, NJ).

### Determination of mRNA levels

Total RNA was prepared from the liver using TRIZOL reagent (Invitrogen Life Technologies, Carlsbad, CA). Samples were quantified by spectrophotometry, and 1 μg of total RNA was used to generate cDNA by reverse transcription (RT) with a Prime Script RT reagent kit (TaKaRa Bio. Inc., Otsu, Japan) according to the manufacturer's directions. cDNA synthesized from 50 ng of total RNA was subjected to a quantitative real-time polymerase chain reaction (PCR) as described previously [18] with an ABI PRISM 7000 Sequence Detector (Applied Biosystems, Foster City, CA) using Premix Ex Taq reagent (TaKaRa Bio Inc.) for the TaqMan probe-based method or SYBR Premix Ex Taq reagent (TaKaRa Bio Inc.) for the intercalation reaction with SYBR Green I according to the manufacturer's specifications. The TaqMan probes and primers for rat *fatty acid synthase *(FAS, NM_017332) and rat *β-actin *(NM_031144), rat *carnitine palmitoyltransferase 1a *(CPT1a, NM_031559) [17], rat *aryl hydrocarbon receptor (*AhR, NM_013149) [21], rat *CYP1A1 *(NM_012540), rat *CYP1A2 *(NM_012541), rat *CYP2B1 *(NM_001134844), rat *CYP3A1 *(NM_173144), and rat *CYP3A2 *(NM_153312), rat *CYP2C11 *(NM_019184), rat *CYP4A1 *(NM_175837), rat *CYP4A2 *(NM_001044770), rat *CYP4A3 *(NM_175760), rat *CPR *(NM_031576), rat constitutive androstane receptor (CAR, NM_022941), rat pregnane X receptor (PXR, NM_052980), and rat retinoid X receptor (RXR, NM_012805) [19], were as reported previously, and those for rat *CYP2E1 *(NM_031543) (assay identification number Rn00580624_ml) and rat *β-actin *(Rn00667869_ml) were assay-on-demand gene expression products (Applied Biosystems). The primers for rat *aryl hydrocarbon nuclear translocator *(ARNT, NM_012780) were 5'-GCACCCAGATGATGTGGATA-3' (forward) and 5'-ATTCCTGCATCTGTTCCTCAA-3' (reverse). The thermal profiles was as follows: 10 sec at 95°C, then a two-step PCR for 40 cycles of 95°C for 5 sec followed by 60°C for 30 sec. β-Actin was used to normalize gene expression in all samples. Fold-increase values were calculated by subtracting the mean difference of gene and β-actin cycle threshold Ct numbers for each treatment group from the mean difference for the vehicle-treated group and raising the difference to the power of 2 (2^-ΔΔCt^).

### Resorufin assay

The activities of ethoxyresorufin *O*-deethylase [reported to prefer CYP1A1 [22]] and methoxyresorufin *O*-demethylase [reported to prefer CYP1A2 [22]] were measured fluorometrically by the production of resorufin using a Wallac 1420 ARVO plate reader (PerkinElmer Inc., Wellesley, MA) with excitation and emission wavelengths of 550 nm and 590 nm, respectively. The incubation mixtures contained Tris-HCl (100 mM, pH 7.4), MgCl_2 _(3.3 mM), EDTA (1 mM), glucose-6-phosphate (3.3 mM), NADP (1.3 mM), glucose-6-phosphate dehydrogenase (0.4 U/ml), ethoxyresorufin or methoxyresorufin (5 μM) and microsomal protein (10 μg/ml). NADPH was produced using the GENTEST NADPH regenerating system (BD Biosciences, Woburn, MA). The mixtures were incubated for 10 min at 37°C. The rate at which resorufin was produced was determined by comparison to the fluorescence of known amounts of resorufin.

### Statistics

Values are expressed as means ± standard errors. The data were analyzed by ANOVA unless stated otherwise. Fisher's Protected Least Significant Difference test was used to determine the significance of differences among the groups. The level of statistical significance was set at *p *< 0.05.

## Results

### Effects of co-treatment with dietary inulin and fluvastatin on biomarkers of metabolic disease in rats fed the HF diet

**Figure 1:**
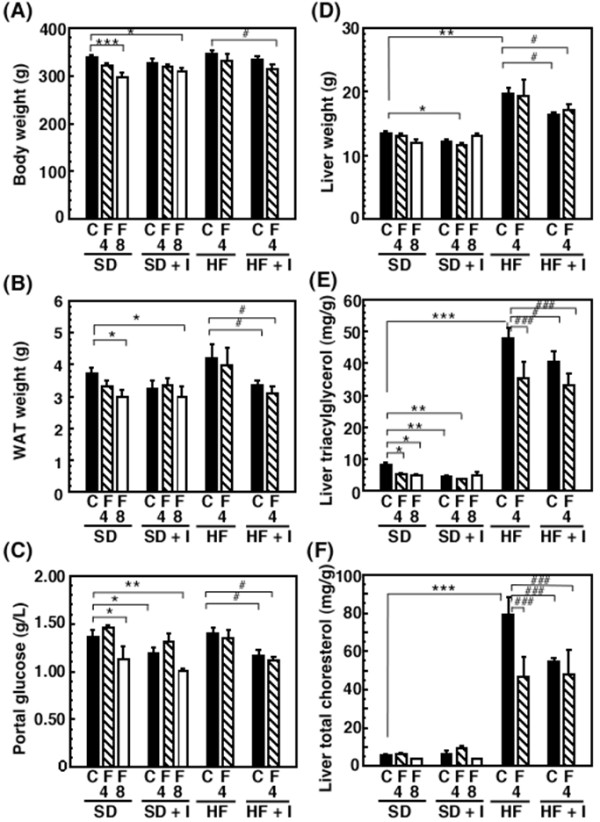
**Effects of synthetic inulin and fluvastatin on body and tissue weights, portal glucose levels, and hepatic lipid profiles in rats fed a standard (SD), inulin-supplemented standard (SD + I), high-fat and high-sucrose (HF), or inulin-supplemented high-fat and high-sucrose (HF + I) diet**. Rats (7 weeks of age) were fed the SD, SD + I, HF, or HF + I diet for 1 week, and then administered fluvastatin (0, 4, or 8 mg/kg) with the diet daily. The rats were sacrificed 14 days after starting the administration of fluvastatin. Values are the mean ± S.E. of 4 determinations. *p < 0.05, **p < 0.01, ***p < 0.001 versus SD diet-fed control rats; # *p *< 0.05, ##*p *< 0.01, ###*p *< 0.001 versus HF diet-fed control rats. C, vehicle; F, fluvastatin; 4, 4 mg/kg; 8, 8 mg/kg.

**Figure 2:**
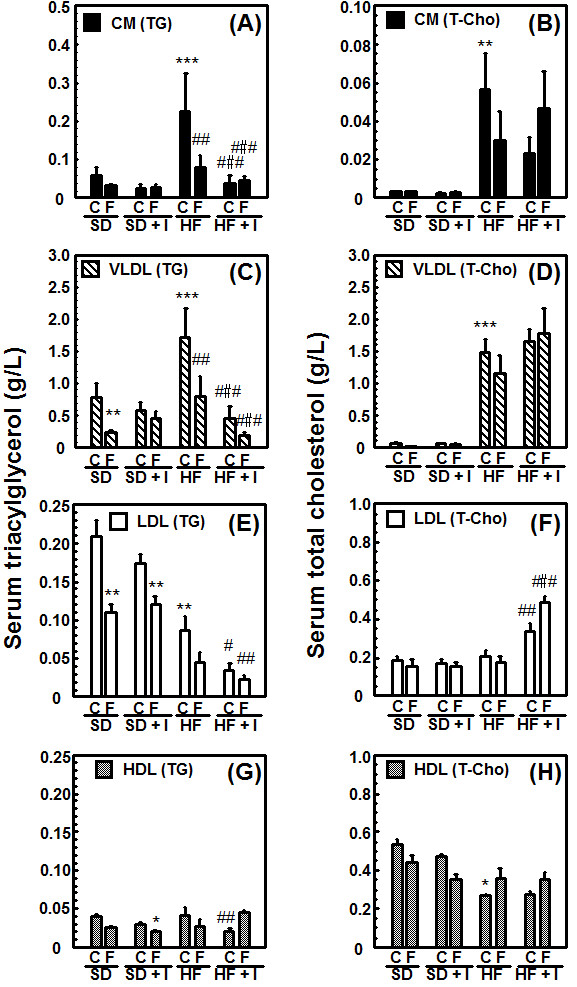
**Comparison of effects of synthetic inulin and fluvastatin on lipid profiles in circulating serum lipoproteins in rats fed a standard (SD), inulin-suplemented standard (SD + I), high-fat and high-sucrose (HF), or inulin-supplemented high-fat and high-sucrose (HF + I) diet**. Rats (7 weeks of age) were fed the SD, SD + I, HF, or HF + I diet for 1 week, and then administered fluvastatin (0 or 4 mg/kg) with the diet daily. The rats were sacrificed 14 days after starting the administration of fluvastatin. Values are the mean ± S.E. of 4 determinations. *p < 0.05, **p < 0.01, ***p < 0.001 versus SD diet-fed control rats; # *p *< 0.05, ##*p *< 0.01, ###*p *< 0.001 versus HF diet-fed control rats.

**Figure 3:**
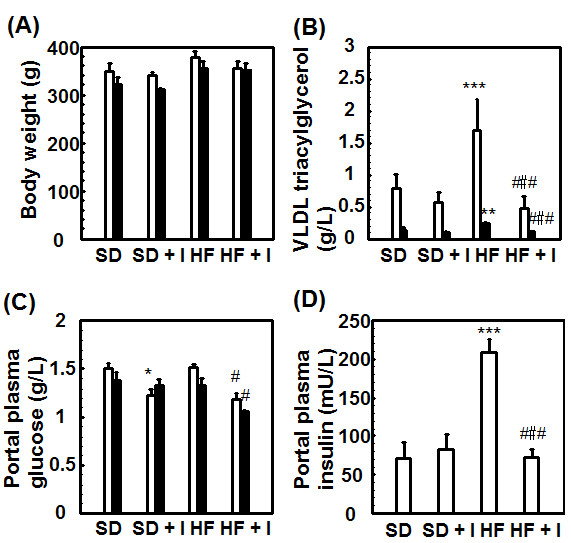
**Comparison of effects of fasting and non-fasting on body weights, circulating blood VLDL triacylglycerol levels, and portal plasma glucose levels in rats fed the SD, SD + I, HF, or HF + I diet**. Rats (7 weeks of age) were fed each diet for 3 weeks. The rats were sacrificed about 4 h after removal of the diet (non-fasting) or after starvation for 16 h. Values are the mean ± S.E. of 4 determinations. *p < 0.05, ***p < 0.001 versus SD diet-fed control rats; # *p *< 0.05, ###*p *< 0.001 versus HF diet-fed control rats. Open bar, non-fasting animals; closed bar, fasting animals.

Rats fed the HF diet for 3 weeks showed fatty livers, which were not associated with an increase in serum aspartate aminotransferase, alanine aminotransferase, and γ-glutamyl transpeptidase levels (data not shown), in addition to an increase in serum triacylglycerol and total cholesterol levels, portal plasma insulin levels, and epididymal WAT and liver weights (Figures 1, 2, 3). Body weight (345.4 ± 7.2 g) and epididymal WAT weight (4.21 ± 0.42 g) of the HF group had a tendency to increase compared to those of the SD group (336.3 ± 7.0 g and 3.72 ± 0.16 g, respectively) and liver weight of the HF group (19.6 ± 1.1 g) significantly increased compared to that of the SD group (13.4 ± 0.4 g) (Figure 1), while the food intake of the HF group (20.6 ± 0.4 g/day) tended to be lower than that of the SD group (22.0 ± 0.7 g/day). The fact that there were no significant rises in body weight of animals fed a high-sugar and high-fat diet has been reported also in monkeys [23]. Consumption of the synthetic inulin for 3 weeks in the HF group suppressed the increase in the weights of the epididymal WAT and liver, hepatic levels of triacylglycerol and cholesterol, serum lipoprotein triacylglycerol levels, especially very low density lipoprotein (VLDL) triacylglycerol levels, and portal plasma insulin levels, but not serum total cholesterol levels (Figures 1, 2, 3). In addition, the intake of dietary inulin reduced portal glucose levels in rats fed not only the HF diet but also the SD diet (Figure 1C). Effect of synthetic inulin on portal plasma glucose levels in the fasting HF rats were similar as that in the non-fasting HF rats (Figure 3). Furthermore, the increase in serum VLDL triacylglycerol levels in the HF group and its suppression by the intake of synthetic inulin were found in the fasting rats as well as in the non-fasting rats (Figure 3). The portal plasma glucose levels in the non-fasting HF group were almost the same as those in the non-fasting SD group (Figure 1). The observation may result from the fact that the portal plasma insulin levels in the HF group markedly increased compared to those in the SD group (Figure 3C). Since rats fed the HF diet and treated with fluvastatin at 8 mg/kg/day died within 2 weeks [20], we examined the effect of fluvastatin at 4 mg/kg/day (*per os*). Fluvastatin given at 4 mg/kg/day for 2 weeks, as a component of the diet, also suppressed serum triacylglycerol levels and hepatic triacylglycerol and total cholesterol levels, but not serum total cholesterol levels, in rats fed the HF diet (Figures 1 and 2). As sown in Figure 1, the extent of the reduction in epididymal WAT weights by fluvastatin was less than that by synthetic inulin at the concentration of 5%, which has been reported to suppress the development of fatty liver [15]. Unexpectedly, co-treatment with inulin and fluvastatin (4 mg/kg) slightly reduced body weights and had a tendency to synergistically or additively suppress the change in epididymal WAT weights and serum triacylglycerol levels (Figures 1 and 2).

**Figure 4:**
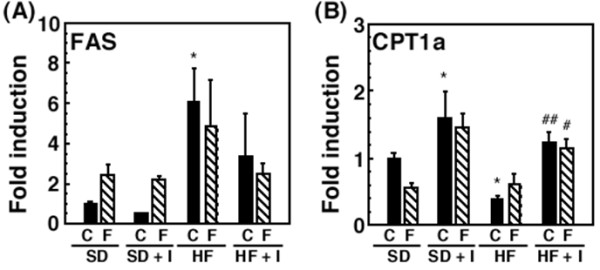
**Liver gene expression [FAS mRNA (A) and CPT1a mRNA (B)] in rats in response to 3-week SD, SD + I, HF, or HF + I diets with or without fluvastatin (0, 4 or 8 mg/kg/day)**. Rats (7 weeks of age) were fed the SD, SD + I, HF, or HF + I diet for 1 week, and then administered fluvastatin (0, 4, or 8 mg/kg) with the diet daily. The rats were sacrificed 14 days after starting the administration of fluvastatin. Values are the mean ± S.E. of 4 determinations. Fold-increase is expressed by taking the control values obtained from SD-fed animals as 1.0. *p < 0.05, **p < 0.01, ***p < 0.001 versus SD diet-fed control rats; # *p *< 0.05, ##*p *< 0.01, ###*p *< 0.001 versus HF diet-fed control rats.

Next, to investigate the mechanism behind the anti-hyperlipidemic effects of dietary inulin, we examined effects on the hepatic gene expression of enzymes such as fatty acid synthase (FAS) and carnitine palmitoyltransferase Ia (CPT1a), involved in the synthesis of fatty acid and β-oxidation, respectively. Consistent with the increase in serum and hepatic triacylglycerol levels, rats fed the HF diet for 3 weeks showed a significant elevation in hepatic FAS mRNA levels and a significant decrease in CPT1a mRNA levels (Figure 4). Dietary inulin had a tendency to suppress the up-regulation of FAS mRNA expression in rats fed the HF diet and suppressed the down-regulation of CPT1a mRNA expression, while fluvastatin had a tendency to suppress both. The co-treatment was not additive or synergistic (Figure 4).

### Nutritional status affects the hepatic expression of drug-metabolizing phase I enzymes in rats

**Figure 5:**
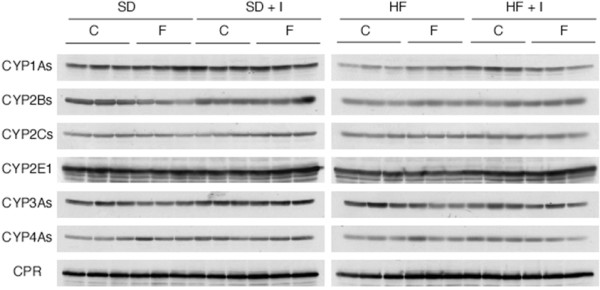
**Effects of synthetic inulin and fluvastatin on expression of cytochrome P450 proteins [western blot] in SD or HF diet-fed rats**. Rats (7 weeks of age) were fed the SD, SD + I, HF, or HF + I diet for 1 week, and then administered fluvastatin (0 or 4 mg/kg) with the diet daily. The rats were sacrificed 14 days after starting the administration of fluvastatin. The microsome proteins (20 μg/lane) were prepared and subjected to immunoblot analysis.

**Figure 6:**
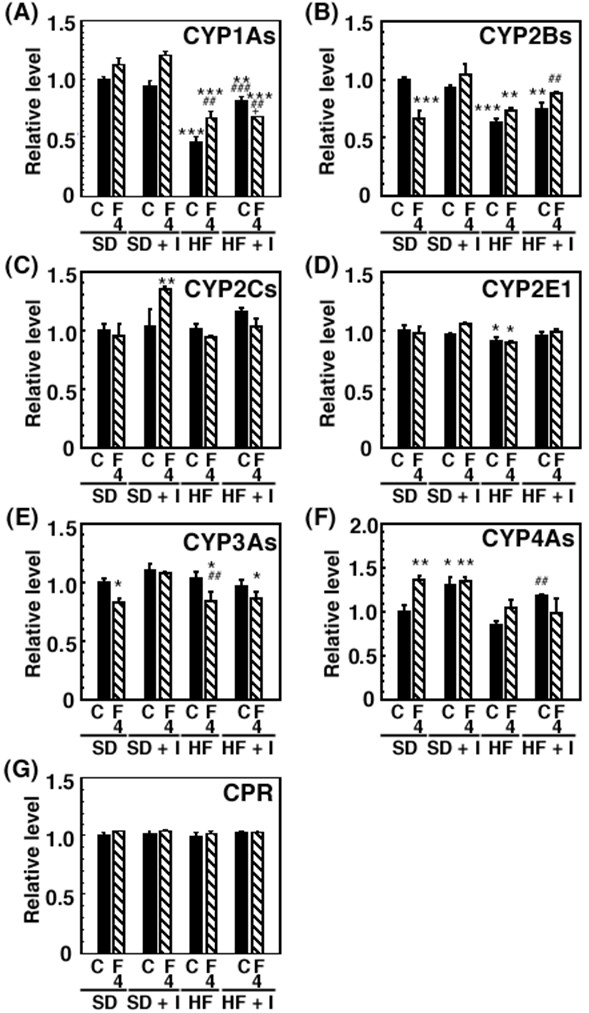
**Effects of synthetic inulin and fluvastatin on expression of cytochrome P450 proteins [levels] in SD or HF diet-fed rats**. Rats (7 weeks of age) were fed the SD, SD + I, HF, or HF + I diet for 1 week, and then administered fluvastatin (0 or 4 mg/kg) with the diet daily. The rats were sacrificed 14 days after starting the administration of fluvastatin. The microsome proteins (20 μg/lane) were prepared and subjected to immunoblot analysis. The signal intensities were determined with CS analyzer (ATTO Densitograph Software Library). Relative levels are expressed by taking the control values obtained from SD-fed control animals as 100. Values are the mean ± S.E. of 3 determinations. *p < 0.05, **p < 0.01, ***p < 0.001 versus SD-fed control rats; ##p < 0.01, ###p < 0.001 versus HF-fed control rats.

**Figure 7:**
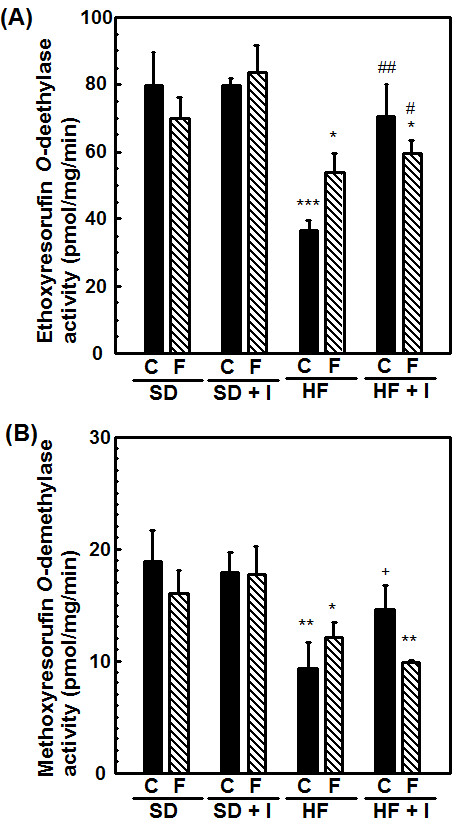
**Effects of synthetic inulin and fluvastatin on hepatic ethoxyresolufin *O*-deethylase (A) and methoxyresolufin *O*-deethylase (B) activities in SD or HF diet-fed rats**. Rats (7 weeks of age) were fed the SD, SD + I, HF, or HF + I diet for 1 week, and then administered fluvastatin (0 or 4 mg/kg) with the diet daily. The rats were sacrificed 14 days after starting the administration of fluvastatin. The microsome proteins were prepared and analyzed for ethoxyresolufin *O*-deethylase and methoxyresolufin *O*-denethylase activities. Values are the mean ± S.E. of 3 determinations. *p < 0.05, **p < 0.01, ***p < 0.001 versus SD diet-fed control rats; #*p *< 0.05, ##*p *< 0.01 versus HF-fed control rats.

Consumption of the HF diet for 3 weeks resulted in a significant decrease in hepatic CYP1A1, CYP1A2, and CYP2E1 mRNA levels to 10 ± 2%, 22 ± 2%, and 37 ± 7%, respectively, of the control values (Table 1). Previously [17], we reported that the administration of clofibrate, a lipid-lowering drug used for controlling the high cholesterol and triacylglycerol levels in blood, (300 mg/kg, *i.p*.) for 5 days reduced the hepatic protein level of CYP1A2 to 20% of the control value and increased the protein levels of CYP4As 4.1-fold. Thus, in this study, we investigated the effect of co-treatment with the synthetic inulin and fluvastatin on changes in the expression of hepatic drug-metabolizing phase I enzymes in rats fed the HF diet. Since fasting alters hepatic expression of the enzymes such as CYP2E1, we focused on alteration in the expression of hepatic drug-metabolizing phase I enzymes in non-fasting rats. As shown in Table 1 and Figures 5 and 6, fluvastatin at 4 mg/kg increased hepatic CYP 1A1 and CYP1A2 mRNA and protein levels in rats fed the SD diet, and slightly restored them in rats fed the HF diet. Dietary inulin recovered the reduced hepatic CYP1A1, CYP1A2, and CYP2E1 mRNA and protein levels and ethoxyresorufin *O*-deethylase and methoxyresorufin *O*-demethylase activities in rats fed the HF diet to near the control values, at the extent of the recovery being higher than with fluvastatin (Table 1 and Figures 5, 6 and 7). However, co-treatment with dietary inulin and fluvastatin did not provide synergistic or additive effects on the recovery of hepatic CYP1A1, CYP1A2, and CYP2E1 mRNA and protein levels and the CYP1A enzyme activities in rats fed the HF diet. Furthermore, we examined effects of nutritional status on transcription factor expression. As shown in Table 1, aryl hydrocarbon receptor (AhR) and aryl hydrocarbon nuclear translocator (ARNT) mRNA levels were reduced in the livers of rats fed the HF diet. The synthetic inulin or fluvastatin alone ameliorated the reduction in AhR and ARNT mRNAs, and the co-treatment did not exert stronger effects than each individual treatment alone, consistent with the alteration in the expression of CYP1A1/2 (Table 1 and Figures 5, 6 and 7).

**Table 1:** Liver gene expression in rats in response to each diet with or without fluvastatin

Gene	SD (fold induction)		Inulin-supplemented SD (fold induction)		HF (fold induction)		Inulin-supplemented HF (fold induction)	
	
	Control	Fluvastatin	Control	Fluvastatin	Control	Fluvastatin	Control	Fluvastatin
*CYP1A1*	1.00 ± 0.44	4.31 ± 0.03**	0.86 ± 0.24	3.13 ± 0.99*	0.10 ± 0.02***	0.24 ± 0.12***	0.31 ± 0.12***	0.15 ± 0.06***

*CYP1A2*	1.00 ± 0.08	1.29 ± 0.06*	1.06 ± 0.09	1.34 ± 0.06*	0.22 ± 0.02***	0.31 ± 0.02***	0.52 ± 0.06***,^#^	0.57 ± 0.03**,^#^

*CYP2B1*	1.00 ± 0.28	1.43 ± 0.39	0.89 ± 0.20	0.83 ± 0.03	0.59 ± 0.09	0.68 ± 0.09	0.57 ± 0.15	0.23 ± 0.05*

*CYP2C11*	1.00 ± 0.04	0.82 ± 0.04	0.81 ± 0.15	0.85 ± 0.08	0.83 ± 0.05	0.76 ± 0.09	0.62 ± 0.15*	0.58 ± 0.03*

*CYP2E1*	1.00 ± 0.06	1.35 ± 0.12	1.43 ± 0.15**	1.47 ± 0.11*	0.37 ± 0.07**	0.84 ± 0.14^#^	0.89 ± 0.10^#^	0.44 ± 0.04*

*CYP3A1*	1.00 ± 0.09	0.81 ± 0.13	1.06 ± 0.05	0.88 ± 0.08	0.77 ± 0.11	0.91 ± 0.19	0.82 ± 0.07	0.54 ± 0.04*

*CYP3A2*	1.00 ± 0.09	0.81 ± 0.07	1.20 ± 0.10	0.87 ± 0.07	0.72 ± 0.09	0.78 ± 0.15	0.74 ± 0.05	0.68 ± 0.07*

*CYP4A1*	1.00 ± 0.06	1.28 ± 0.13	1.76 ± 0.73	1.20 ± 0.10	0.53 ± 0.04	0.64 ± 0.20	1.43 ± 0.28^#^	1.12 ± 0.17

*CYP4A2*	1.00 ± 0.14	2.45 ± 0.54*	2.94 ± 0.54*	1.46 ± 0.19	0.42 ± 0.28	0.39 ± 0.27	1.00 ± 0.24	0.34 ± 0.01

*CYP4A3*	1.00 ± 0.08	1.08 ± 0.14	1.24 ± 0.40	0.80 ± 0.08	0.53 ± 0.03	0.55 ± 0.13	1.33 ± 0.29	1.01 ± 0.08

*CPR*	1.00 ± 0.09	1.17 ± 0.13	1.17 ± 0.07	1.45 ± 0.12	1.20 ± 0.12	1.03 ± 0.05	1.23 ± 0.15	1.19 ± 0.21

*CAR*	1.00 ± 0.10	0.74 ± 0.15	1.51 ± 0.20	1.31 ± 0.24	1.44 ± 0.33	1.26 ± 0.36	1.51 ± 0.20	0.47 ± 0.21

*PXR*	1.00 ± 0.04	0.81 ± 0.06	1.10 ± 0.16	1.08 ± 0.11	0.85 ± 0.10	0.86 ± 0.10	0.93 ± 0.08	0.60 ± 0.05*

*RXR*	1.00 ± 0.05	0.76 ± 0.03	0.98 ± 0.06	0.88 ± 0.08	1.08 ± 0.04	0.92 ± 0.06	1.13 ± 0.06	1.06 ± 0.26

*AhR*	1.00 ± 0.20	0.74 ± 0.07	1.09 ± 0.26	1.04 ± 0.16	0.52 ± 0.03*	1.10 ± 0.17	1.02 ± 0.09	0.68 ± 0.13

*ARNT*	1.00 ± 0.06	0.86 ± 0.05	0.91 ± 0.02	0.81 ± 0.03	0.69 ± 0.09***	0.76 ± 0.05**	0.80 ± 0.06	0.74 ± 0.04*

*p < 0.05, **p < 0.01, ***p < 0.001 versus SD-fed animals; #p < 0.05 versus HF-fed animals.

Rats were fed the SD-, inulin-supplemented SD-, HF-, or inulin-supplemented HF diets for 1 week, continued to consume each diet containing fluvastatin (0 or 4 mg/kg/day) for another 2 weeks, and then sacrificed. Relative levels were expressed by taking the control values obtained from SD-diet-fed rats as 1.00. Values are the means ± S.E. of 4 determinations in each group.

## Discussion

**Figure 8:**
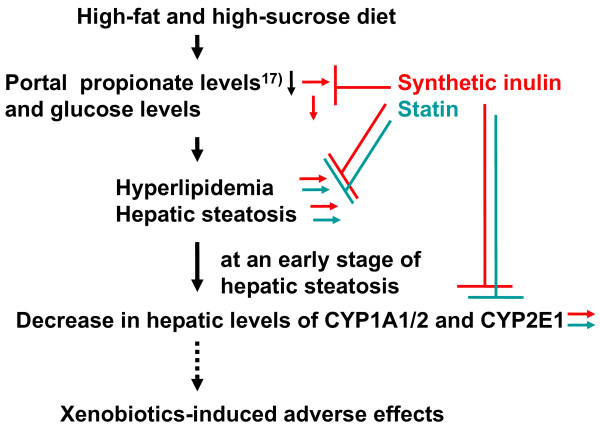
**Proposed scheme of hyperlipidemia and hepatic steatosis in rats fed the HF diet, xenobiotics-induced adverse effects, and suppression by synthetic inulin and statin**.

Nutritional factors such as starvation, fasting, and a high-lipid diet, and pathophysiological factors such as diabetes have been reported to affect liver drug-metabolizing phase I enzymes, leading to the altered hepatic metabolism of drugs, carcinogens, steroid hormones, and fatty acids [1-4,11,24-26]. The accumulation of triacylglycerols in the liver, defined as hepatic steatosis, is proposed to be the first stage of more severe liver diseases such as nonalcoholic steatohepatitis, which shows histological signs of fibrosis and necroinflammation, through cirrhosis, terminal liver failure, and hepatocellular carcinoma [11]. In the previous studies [17,19], we reported the decrease in portal plasma propionate levels and severe hepatic steatosis in rats fed the HF diet. Inulin is fermented by colonic microflora, and short-chain fatty acids such as acetate, propionate and butyrate are produced and can be absorbed from the colon [14,16]. Propionate is reported to inhibit fatty acid synthesis in rat hepatocytes [27,28] and the reduction in propionate levels in portal plasma may be associated with induction of liver fatty acid synthase. This study demonstrated that in rats fed the HF diet but not the SD diet for 3 weeks, synthetic inulin (5%) decreased the portal plasma glucose levels, and suppressed the triacylglycerol accumulation in blood and liver (Figures 1, 2, 3). In addition, synthetic inulin has been reported to recover the decreased portal plasma propionate levels (Reference [17] and Figure 8). Thus, the recovery of portal plasma propionate levels by synthetic inulin could result in suppression of fatty acid synthesis. These observations were consistent with the alteration in FAS and CPT1a mRNA levels (Figures 1, 2, 3, 4). FAS mRNA levels were elevated in the liver of rats fed the HF diet, and dietary inulin suppressed the elevated mRNA levels of fatty acid synthase, suggesting the suppression of liver *de novo *lipogenesis, but the co-treatment with fluvastatin did not exert stronger effects than each individual treatment alone. In addition, the mRNA expression of the β-oxidation-related enzyme CPT1a was reduced in the livers of HF rats, and recovered to nearly normal levels with the intake of synthetic inulin, indicating the anti-lipogenic action of synthetic inulin (Figures 1, 2, 3, 4 and 8). Since the total cholesterol level in the serum was not suppressed by the synthetic inulin in rats fed the HF diet (Figure 1), we examined the effects of co-treatment with synthetic inulin and fluvastatin. Unexpectedly, an additive or synergistic effect of co-treatment on the suppression of elevated serum and liver total cholesterol levels was not found, while the suppression of elevated serum and liver triacylglycerol levels was slightly stronger than that by each individual treatment alone (Figures 1 and 2). In the previous study [20], we found the severe adverse effects of fluvastatin (8 mg/kg/day) in rats fed the HF diet. Preceding the elevation in serum creatine kinase levels and muscle damage in rats fed the HF diet containing fluvastatin (8 mg/kg/day), we have found that serum AST levels were elevated, but the increase in serum AST levels were not observed in rats fed the HF diet containing fluvastatin (4 mg/kg/day). Although fluvastatin at 4 mg/kg/day for 2 weeks did not change the hepatic levels of its uptake transporter, organic anion transporter 2, in rats fed the HF diet (data not shown), undesirable adverse effects of fluvastatin even at low dose of 4 mg/kg/day may occur for longer term at the lower rate but those of synthetic inulin were not found for 12 weeks [15,17,19].

The elevated expression of CYP2E1, a microsomal oxidase involved in fatty acid ω-oxidation, as well as CYP4A, has been shown to be largely responsible for the pathogenesis of liver disease in patients with nonalcoholic steatohepatitis and plays a key role in the development of liver injury by initiating lipid peroxidation [3-7]. There was the discrepancy in mRNA and protein levels of hepatic CYP2E1, while both hepatic CYP2E1 mRNA and protein levels were reduced in rats fed the HF diet and synthetic inulin tended to recover the levels (Table 1 and Figure 6D). Rat hepatic CYP2E1 has been reported to be regulated at two different levels (a transcriptional level to increase CYP2E1 mRNA expression and a post-transcriptional level to regulate CYP2E1 protein and activity) [29,30]. In rats fed the HF diet, hepatic CYP2E1 regulation may occur mainly at the post-transcriptional level. In our previous study [19], we have demonstrated that the feeding of the HF diet to rats for 8 weeks produces hepatic steatosis, which is associated with the induction of liver injury by xenobiotics such as phenobarbital and dexamethasone via induction of CYP2B and CYP3A expression, but not associated with liver injury via the alteration of hepatic CYP2E1 and CYP4As. The present study demonstrated that feeding the HF diet to rats for 3 weeks reduced CYP2E1 and CYP1A1/2 mRNA and protein levels, and the improvement in nutritional status in the liver caused by the synthetic inulin alone ameliorated the reduction in the expression of CYP2E1 and CYP1A1/2, indicating the reduction to occur in the initial stage in the alteration of drug-metabolizing enzymes in the fatty liver induced by a HF diet.

CYP1 enzymes such as CYP1A1, CYP1A2, and CYP1B1 not only play an important role in the metabolic activation of environmental procarcinogens, but also metabolize large numbers of clinically important drugs such as caffeine and theophiline and several important endogenous substrates such as melatonin, bilirubin, esteron, and estradiol [31-35]. There have been many epidemiological studies on the inducibility of hepatic CYP1A enzymes by diet, and their association with the metabolism of environmental and dietary carcinogens [36-38]. Dietary indolyl glucosinolates and flavonoids have been reported to induce CYP1A expression either through direct interaction with AhR or by augmenting the interaction of AhR with xenobiotic response elements in CYP1A1 and other target genes. Generally, transcriptional up-regulation of drug-metabolizing phase 1 enzymes by xenobiotics occurs via CAR (CYP2B induction), PXR (CYP3A induction), AhR (CYP1A induction), PPARα (CYP4As induction) and Nrf2 (NADPH-quinone oxidoreductase induction). CYP1A2 (a major member of the CYP1A subfamily in rat liver), is partly induced through transactivation mediated by AhR, which is a ligand-activated transcription factor and forms a heterodimer with ARNT. In this study, we demonstrated that in fatty liver caused by feeding a HF diet for 3 weeks, the damage from which occurred prior to nonalcoholic hepatic steatosis, CYP1A1 and CYP1A2 mRNA and protein expression and related metabolic activities of ethoxyresorufin *O*-deethylase and methoxyresorufin *O*-demethylase, respectively, were down-regulated (Table 1 and Figures 5, 6 and 7). Since the AhR mRNA level in the liver of rats fed the HF diet was also reduced, the suppressive effect of the HF diet on the baseline expression of CYP1A1 and CYP1A2 may be AhR-dependent. Synthetic inulin ameliorated the change in the expression of CYP1A1 and CYP1A2 in rats fed the HF diet, in association with a suppression of the development of hepatic steatosis.

## Conclusion

This study indicated (1) that changes in the nutritional status of the liver in rats fed a HF diet may cause adverse effects due to reduced expression of CYP1A1, CYP1A2, and CYP2E1, in addition to adverse effects of lipid-lowering drugs such as fluvastatin and (2) that intake of dietary inulin alone suppressed the development of hepatic steatosis and ameliorated the nutritional status, followed by a suppression of the reduction in hepatic expression of drug-metabolizing enzymes such as CYP1A1, CYP1A2, and CYP2E1, while co-treatment with statin had slightly additive or synergistic effects.

## Abbreviations

AhR: Aryl hydrocarbon receptor; ARNT: Aryl hydrocarbon nuclear translocator; C: Vehicle; CAR: Constitutive androstane receptor; CPR: NADPH-cytochrome P450 reductase; CPT1a: Carnitine palmitoyltransferase 1a; CYP: Cytochrome P450; DP: Degree of fructose polymerization; FAS: Fatty acid synthase; HF diet: High-fat and high-sucrose diet; HF + I diet: Inulin-supplemented HF diet; HMG-CoA reductase: 3-hydroxy-3-methylglutaryl CoA reductase; PCR: Polymerase chain reaction; PXR: Pregnane X receptor; RXR: Retinoid X receptor; SD diet: Standard diet; SD + I diet: Inulin-supplemented SD diet; VLDL: Very low density lipoprotein; WAT: White adipose tissue.

## Competing interests

The authors declare that they have no competing interests.

## Authors' contributions

JS has made substantial contributions to conception and design, has been involved in drafting the manuscript, and has given final approval of the version to be published. SS has made acquisition of data and analysis and interpretation of data. TW has been involved in revising it critically for important intellectual content. YY has been involved in revising it critically for important intellectual content. AI has been involved in revising it critically for important intellectual content. MM has been involved in drafting the manuscript, and has given final approval of the version to be published. All authors read and approved the final manuscript.
